# Tuning of ionic mobility to improve the resistive switching behavior of Zn-doped CeO_2_

**DOI:** 10.1038/s41598-019-55716-4

**Published:** 2019-12-18

**Authors:** Shania Rehman, Honggyun Kim, Muhammad Farooq Khan, Ji-Hyun Hur, Anthony D. Lee, Deok-kee Kim

**Affiliations:** 1 Department of Electrical Engineering, Sejong University, Seoul, 05006 Republic of Korea; 2 Department of Mechanical Engineering Technology, Farmingdale State College, Farmingdale New York, 11735 USA

**Keywords:** Electronic devices, Information storage

## Abstract

Correlation between the resistive switching characteristics of Au/Zn-doped CeO_2_/Au devices and ionic mobility of CeO_2_ altered by the dopant concentration were explored. It was found that the ionic mobility of CeO_2_ has a profound effect on the operating voltages of the devices. The magnitude of operating voltage was observed to decrease when the doping concentration of Zn was increased up to 14%. After further increasing the doping level to 24%, the device hardly exhibits any resistive switching. At a low doping concentration, only isolated V_o_ existed in the CeO_2_ lattice. At an intermediate doping concentration, the association between dopant and V_o_ formed (*Zn*, *V*_*o*_)^×^ defect clusters. Low number density of these defect clusters initially favored the formation of V_o_ filament and led to a reduction in operating voltage. As the size and number density of (*Zn*, *V*_*o*_)^×^ defect clusters increased at a higher doping concentration, the ionic conductivity was limited with the trapping of isolated V_o_ by these defect clusters, which resulted in the diminishing of resistive switching. This research work provides a strategy for tuning the mobility of V_o_ to modulate resistive switching characteristics for non-volatile memory applications.

## Introduction

As existing semiconductor technologies are approaching their physical scaling limits, a new memristive device concept^1,2^ has gained great attention for its use in future highly scalable nonvolatile memories. The switching mechanism in resistive random-access memory (RRAM) is governed by the oxide ion migration and the formation of oxygen vacancy (V_o_) filament within the metal oxide thin films. The ionic migration is driven by the electric field induced drift motion and concentration gradient dependent diffusion^3^. This drift/diffusion of V_o_ is supposed to play a key role in determining the ultimate resistive switching behavior of the devices. The ionic diffusion coefficient is expressed as $D=[{V}_{o}^{\mathrm{.}.}]\gamma {a}^{2}\theta {e}^{(-{E}_{m}/{k}_{B}T)}$^4^, where $[{V}_{o}^{\mathrm{}..}]$ is the concentration of oxygen vacancies, γ is a constant, a is the jump distance, θ is the attempt to escape frequency, E_m_ is the oxide ion migration energy barrier, k_B_ is the Boltzmann constant, and T is the temperature. The above expression makes it clear that the diffusion coefficient depends on $[{V}_{o}^{\mathrm{}..}]$ the mobility of oxygen ion via ${e}^{(-{E}_{m}/{k}_{B}T)}$. The mobility of the oxygen ions is directly proportional to the ionic conductivity of the oxygen ions. Hence, it can be assumed that the mobility or ionic conductivity of oxygen ions and the concentration of oxygen vacancies are the key parameters to control the resistive switching behavior in RRAM.

The ionic conductivity of pure CeO_2_ used in this study is not very high because of the low concentration of oxygen vacancies^5^. However, the exceptional sensitivity of ionic conductivity of doped CeO_2_ associated with doping level have been demonstrated^6,7^. The ionic conductivity of CeO_2_ can be modulated by doping it with lower valency (bivalent or trivalent) cations. Theoretical studies indicate that Ce^4+^/Ce^3+^ reduction is greatly enhanced when the CeO_2_ is doped with bivalent or trivalent oxides^6^. When CeO_2_ is doped with bivalent oxide, Ce (IV) atoms of host lattice are replaced with bivalent cations, and an O vacancy is formed in order to compensate the created charge. These created vacancies make the diffusion of O ions easier, and increase ionic conductivity. The formation of the oxygen vacancy results in the reduction of two neighboring Ce ions from Ce^4+^ to Ce^3+ ^^7,8^. This increase in concentration of oxygen vacancies and their mobility on doping with bivalent dopant may control the characteristics of memory devices such as switching speed, operating voltage, and the R_on_/R_off_ ratio. Although the resistive switching behavior of CeO_2_ films has already been investigated^9–11^, the previous studies on the resistive switching characteristics of CeO_2_ has encountered the demerits of high operating voltage^12^ and a low memory window^13^.

Different strategies such as doping^14^ or interface engineering which includes the introduction of a CeO_x_/silicon (Si) interface^15^, ZrO_y_ interfacial layer^10^ or the use of reactive metal electrodes^16^ was adopted for the creation of oxygen vacancies in CeO_2_ films to reduce the operating voltages and improve endurance. In this study, we have utilized a different approach to modulate the level of oxygen stoichiometry and defects in the CeO_2_. It is known that bivalent dopants are more efficient for obtaining the lower reduction energy because the bivalent dopant may introduce twice the number of oxygen vacancies in host CeO_2_ lattice as compared to trivalent dopants at the same doping level^6^ (Supporting information S1). We chose Zn^2+^ (0.091 nm) as a bivalent dopant having comparable ionic radii with Ce^4+^ (0.097 nm), because it not only increases the reducibility of CeO_2_^17,18^, but also is economic and easily available compared to high ionic rare earth metal dopants such as Sm^3+^ and Ga^3+^. The Zn doping level in this study was much higher as compared to previous studies, where doping was initiated by using an electric field stimulated diffusion of metal ions from an inserted metal layer^10,19,20^. However, the doping level was kept below the solubility limit^21^ (20–30%) to avoid the complexity of the secondary phase evolution of ZnO in CeO_2_. Based on previous studies^6,22,23^, it is clear that the dopant incorporation in the CeO_2_ lattice has a significant influence on the transport properties of O ions. The interactions between dopants and oxygen vacancies at higher doping levels play an important role in determining the mobility of oxygen ions. The effect of the defect interaction with oxygen vacancies on the resistive switching mechanism has rarely been reported before. At low doping levels, isolated V_o_ are created, which results in an increase of mobility of the oxygen ion. At medium doping levels, defect associates or clusters are formed with certain binding or association energy because of interactions between dopants and oxygen vacancies, but the number density of these defect is very low at medium doping levels to affect the mobility of oxygen ions. At higher doping concentrations, the number density of these defect associates increases and prevent oxygen vacancy from being mobile, and they consequently affect the ionic conductivity^24–26^. There are two major factors that determine the association energy of a dopant-oxygen vacancy cluster. The first factor is the Coulombic interaction that corresponds to the electrostatic attraction among the dopant ions and oxygen vacancies, and the second is the elastic interactions that refers to the size mismatch of dopants as compared to the host lattice^27–29^. Hence, the valence and ionic radius of the dopant cations play a key role in modulating the electrical conductivity of doped CeO_2_. The defect chemistry of Zn-doped CeO_2_ is given in the Supplementary information S2.

## Experimental Details

Au was deposited as the bottom electrode by an e-beam evaporator with a thickness of 70 nm. CeO_2_ and ZnO targets (Superconductor Materials (SCM), USA) were used for the deposition of the active layer in the RF sputtering unit. Prior to the deposition of CeO_2_, the sputtering chamber was evacuated down to a pressure level of 2 × 10^−6^ Torr. During the deposition, working pressure inside the chamber was 22 mTorr. Ar and O_2_ gases with the flow rates of 14 sccm and 2 sccm were introduced into the chamber. The RF-power of the CeO_2_ target was kept at 150 W. The RF power of the ZnO target was varied from 35 W and 45 W to 55 W to modulate the doping level in different Zn-doped CeO_2_ samples. According to the doping levels determined by XPS, the samples are labelled as 6ZnCeO_2_, 14ZnCeO_2_ and 24ZnCeO_2_ for Zn-doped CeO_2_ samples deposited by the ZnO sputtering target with RF power of 35 W, 45 W, and 55 W, respectively. After deposition, samples were annealed at 500 °C for 20 minutes in an Ar environment to allow uniform doping. Finally, the top electrode of Au was deposited by an e-beam evaporator with a thickness of 70 nm and an area of 75 × 75 μm^2^ using a shadow mask. The surface composition and chemical properties were analyzed by a Thermo Fisher Scientific (with K-alpha X-ray source) X-ray photoelectron Spectroscopy (XPS). The Raman spectra were obtained using a Renishaw micro-spectrometer with a laser wavelength of 514 nm at room temperature. The spot size was ~1 µm and the power was maintained at ~1.0 mW to reduce the heating effects. The electrochemical impedance spectroscopy was performed using a ZIVE SP2 electrochemical workstation (WonATech Co., Ltd, Republic of Korea). The measured frequency ranged from 0.1 Hz to 1 MHz under a bias voltage of 10 mV. The electrical characteristics were measured using an Agilent B1500 semiconductor characterization system at room temperature.

## Results and Discussion

**Figure 1:**
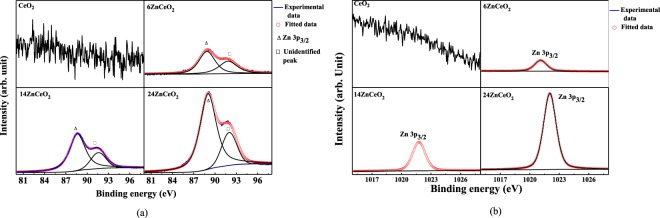
(**a**) High resolution XPS spectra of Zn 3p_3/2_ in un-doped and Zn-doped CeO_2_ samples. (**b**) High resolution XPS spectra of Zn 3p_3/2_ in un-doped and Zn-doped CeO_2_ samples.

XPS is utilized to determine the elemental composition and valence states of the Zn-doped CeO_2_ samples. The detailed survey XPS spectra of un-doped CeO_2_ and Zn-doped CeO_2_ are shown in Figure S3(a). The spectra revealed the existence of characteristic peaks of Ce, Zn, and O. In order to calculate the elemental composition and identification of chemical states, the high-resolution O 1s, Zn 2d, and Ce 3d core level spectra are discussed in more detail below. Figure 1(a,b) display the Zn 3d XPS spectra for un-doped and Zn-doped CeO_2_ in the binding energy range from 80 eV to 98 eV and from 1015 eV to 1028 eV with different concentrations of Zn controlled by changing the RF power of the ZnO target from 35 W to 55 W with an increment of 10 W. The spectra in Fig. 1(a) is de-convoluted into two peaks. On the other hand, the spectra in Fig. 1(b) is fitted with one peak. The characteristic peaks of Zn are observed at 89 eV in Fig. 1(a), and 1022 eV in Fig. 1(b), respectively. This spectrum confirmed the Zn doping in CeO_2_. The Zn^2+^ concentration in each sample was estimated by adding the areas under the curves of the 89 eV and 1022 eV peaks, and dividing by the sum of the areas of all characteristic peaks multiplied by their cross-section of Ce^3+^, Ce^4+^, Zn^2+^ and O^2+^ in the spectra.

**Figure 2:**
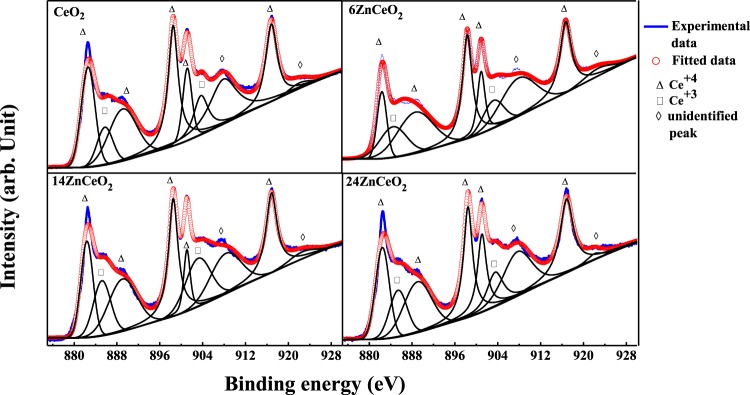
(**a**) High resolution XPS spectra of Ce 3d in un-doped and Zn-doped CeO_2_ samples.

In order to analyze the effect of dopant on the surface chemistry and estimate the relative fraction of Ce^4+^ and Ce^3+^ oxidation states in the Zn-doped CeO_2_ samples, the Ce 3d spectra was de-convoluted into eight peaks as shown in Fig. 2. The peaks at 885 eV and 903.5 eV are assigned to Ce^3+^, while 882 eV, 898 eV, and 916.35 eV are attributed to the Ce^4+^ state^30^. The coexistence of Ce^4+^ and Ce^3+^ ions can be seen in each sample. The relative concentration of the Ce^3+^ species in each sample is calculated by dividing the sum of the integrated areas of the Ce^3+^ peaks to the total area of all the peaks (Ce^3+^ and Ce^4+^ species) in the spectrum. The calculated concentration of the Ce^3+^ ions was 14%, 21%, 26%, and 22% in un-doped CeO_2_, 6ZnCeO_2_, 14ZnCeO_2_ and 24ZnCeO_2,_ respectively. The analysis showed that the Ce^3+^ concentration was increasing in the samples with the increase in the doping concentration. It has been reported that the presence of Ce^3+^ ions in the CeO_2_ is linked with the formation of oxygen vacancies^8^. It is described in the previous section that when the CeO_2_ is doped with bivalent ions, the Ce^4+^/Ce^3+^ reduction is greatly enhanced. When the Zn dopant substitute was Ce^4+^, an O vacancy formed inside the CeO_2_ lattice. The formation of the oxygen vacancy resulted in the reduction of two neighboring Ce ions from Ce^4+^ to Ce^3+^. Thus, the systematic increase in Ce^3+^ content in the 6ZnCeO_2_ and 14ZnCeO_2_ was an indication of more oxygen vacancies on increasing the doping concentration. However, the decrease in Ce^3+^ content was observed in the 24ZnCeO_2_ sample on increasing the dopant concentration. The slightly decreased Ce^3+^ concentration in 24ZnCeO_2_, which is unlike other doping concentrations indicated that there was a saturation of isolated oxygen vacancies at this point. At low doping concentrations, association between Ce^4+^ and V_o_ was strong, which resulted in the enhancement of Ce^3+^. As the doping level increased, the association between dopant and V_o_ became stronger, which resulted in the formation of (*Zn*, *V*_*o*_)^×^ defect clusters, and the preferred substitutional position of dopant was in the defect cluster (*Zn*, *V*_*o*_)^×^. This resulted in a decrease in the reduction process of Ce^4+^ to Ce^3+^ by an interaction with nearby V_o_^26^.

The high-resolution O 1s core-level spectra is shown in Figure S3(b) which is de-convoluted into two peaks for further analysis. The peaks in the range of 531.0–532.6 eV can be attributed to the surface oxygen species adsorbed on the oxygen vacancies (i.e., O^−^, OH^−^). However, the binding energy at 529.4 eV was assigned to lattice oxygen^31^. The spectrum was composed of lattice oxygen and chemisorbed oxygen species. For analysis, we only considered the contribution of the peak associated with lattice oxygen. As it can be seen in Figure S3(b), the intensity of the peak is reduced on increasing the doping concentration. As previously discussed, increasing the doping concentration creates more oxygen vacancies. This decrease in the intensity of the peak is associated with the formation of more oxygen vacancies on increasing the doping concentration.

**Figure 3:**
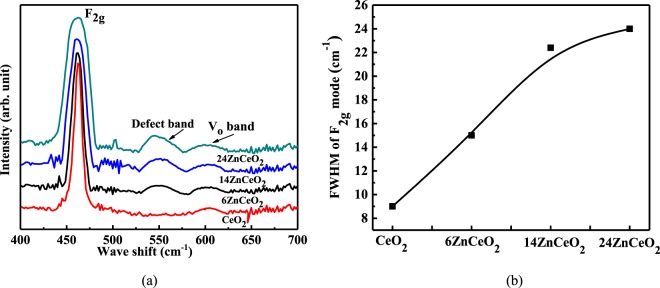
(**a**) Raman spectra of un-doped and Zn-doped CeO_2_ samples. (**b**) Plot of variation of FWHM of F_2g_ mode in undoped and Zn doped CeO_2_ samples.

A Raman spectroscopy was employed to study the relative change in vibrational modes and lattice structural characteristics of CeO_2_ as a function of Zn doping. Raman spectroscopy is an efficient technique to study symmetry breaking and defect associates in doped CeO_2_^32^. This technique is very useful to detect the changes in the bonding atmosphere, because it allows a thorough analysis of the Ce–O bonds^32,33^. The excitation laser of wavelength 514 nm can provide information about bulk of Zn-doped CeO_2_^22^. Figure 3 displays the Raman spectrum of Zn-doped CeO_2_ thin films measured in the range of 400 cm^−1^ to 700 cm^−1^. The main Raman-active mode (F_2g_) in a fluorite-type CeO_2_ due to Ce−O stretching vibration, is located around 462 cm^−1 ^^34^. It is considered that the F_2g_ mode is assigned to the symmetric breathing mode of oxygen ions around the Ce cation, and its position is strongly dependent on the Ce (cation)-O (anion) bond strength^35^. As can be seen in the Raman spectrum of the Zn-doped CeO_2_, increasing the doping concentration results in an increase in FWHM and a frequency shift of the F_2g_ peak. This increase in FWHM and a frequency shift are associated with structural disorder induced by the dopant by increasing the dopant concentration^36^.

Additional modes at 555 cm^−1^ and 610 cm^−1^ were also observed in the Raman spectra. A Peak at 590 cm^−1^ originated due to oxygen vacancies and disturbed local symmetry induced by the different sizes of the dopants and a peak at 610 cm^−1^ in CeO_2_ is associated with intrinsic oxygen vacancies^37,38^. In this case, different sizes of Zn^2+^ versus Ce^4+^ cations were responsible to activate the 555 cm^−1^ mode in the doped CeO_2_ samples. The presence of these modes can be associated with the homogeneous incorporation of Zn within the CeO_2_ crystal structure and the formation of oxygen vacancies associated with this phenomenon. The oxygen vacancy peak found in the un-doped CeO_2_, can be associated with the presence of some intrinsic vacancy. As the deposition was performed in a very low O_2_ atmosphere, it may also have contributed to the formation of oxygen vacancies. The enhancement of the 555 cm^−1^ mode with the increase in doping concentration was associated with the increase in oxygen vacancies and associated structural disorder on increasing doping concentration.

**Figure 4:**
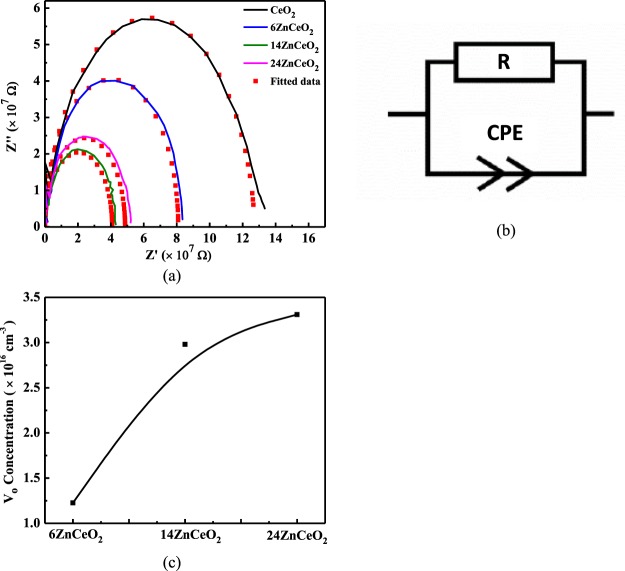
(**a**) Electrochemical impedance spectra of un-doped and Zn-doped CeO_2_ measured in atmosphere at 250 °C. (**b**) Equivalent circuit to analyze the resistance ‘R’ and constant phase element ‘CPE’. (**c**) Variation in concentration of V_o_ at different doping levels.

Electrochemical impedance spectroscopy (EIS) is employed to study the influence of doping on the ionic conductivities of the as-synthesized un-doped and Zn-doped CeO_2_ samples. Generally, for the case of ionic conductivity materials, the EIS mainly consists of three arcs: the high frequency arc, the middle-frequency arc, and the low frequency tail. The high frequency arc, the middle frequency arc, and the low frequency tail correspond to the grain interior, grain boundaries, and electrode contribution to the overall conductivity of the sample^22^. Figure 4(a) shows the typical Nyquist plots for the CeO_2_, 6ZnCeO_2_, 14ZnCeO_2_, and 24ZnCeO_2_ samples obtained in air. These plots, which comprised of one semicircle, were different from the typical Nyquist plots of un-doped and Zn-doped CeO_2_ that consist of two separate semicircles^39^. This difference was assigned to the existence of experimental stray capacitance, which was several orders of magnitude higher than the capacitance of the bulk and grain boundaries of the film^40,41^. Since the existence of stray capacitance makes it difficult to distinguish between the contribution of grain interior and grain boundary, only the additive effect of both resistances can be measured. The equivalent circuit shown in Fig. 4(b) consists of the resistance R and a constant phase element (CPE) in parallel was used for the fitting of Nyquist plot. The CPE is the replacement of ideal capacitor. Mathematically, impedance of the CPE is defined as^42^

1 $$Z=\frac{1}{i{w}^{\alpha }{C}_{\alpha }},$$

where *i*, *w*, and α are $\sqrt{-1}$, angular frequency and a factor associated with the deviation from ideal resistor, capacitance, and inductor. It corresponds to a resistor, a capacitor and an inductor when α = 0, α = 1 and α = −1, respectively. In the actual application of this element, α is defined between 0 and 1, and this element can be assumed a generalization of a conventional capacitor. C_α_ is the constant phase element. The equivalent circuit shown in Fig. 4(b) and corresponding parameters (R and CPE) were obtained by fitting of the experimental data using ZMAN software. According to the fitted results, the values of R and CPE are listed in Table 1 and plotted in Figure S4. As is shown in Table 1 and Figure S4, the resistance of CeO_2_ decreases with the increase in doping concentration up to 14%. With further increase of the doping concentration, a slight increase in the resistance of 24ZnCeO_2_ was observed. At low doping concentrations, dopant cations substitute the Ce^4+^ in the lattice structure, form a solid solution and, increases the concentration of isolated oxygen vacancies. This leads to an increase in the ionic conductivity. At intermediate doping concentrations, association between the dopant and V_o_ forms clusters. Both the size and number density of these clusters increases with the doping concentration. For high doping concentrations, conductivity is reduced due to decreasing mobility of isolated V_o_ by the increased number density of the clusters. The isolated V_o_ gets trapped in these (*Zn*, *V*_*o*_)^×^ defect clusters and affects the conductivity of the heavily doped sample^8,43,44^.

**Table 1:** Parameters extracted from the fitted data using experimentally obtained EIS spectra with equivalent circuit, for undoped and doped CeO_2_ with different doping levels.

Composition	R (Ω)	CPE (F.S^(α −1)^)	α
CeO_2_	1.27 × 10^7^	5.48 × 10^−10^	0.964
6ZnCeO_2_	8.03 × 10^7^	4.44 × 10^−10^	0.934
14ZnCeO_2_	4.08 × 10^7^	2.63 × 10^−10^	0.927
24ZnCeO_2_	4.81 × 10^7^	3.33 × 10^−10^	0.932

The performance of memory devices is associated with the movement of oxygen ions through V_o_, under the influence of external electric field. The resistive switching characteristics of these devices are strongly affected by the concentration of the isolated oxygen vacancies or clustered oxygen vacancies. The concentration of isolated V_o_ can be obtained by utilizing the chemical capacitance C_chem_ extracted by the impedance spectroscopy^42^. The relationship between the concentration of V_o_ in the doped CeO_2_ film and the C_chem_ extracted by the impedance spectroscopy is explored by D. Chen *et al*.^42^. The chemical capacitance is defined as a measure of material’s chemical storage ability under the influence of applied potential as follows:

2 $${{\rm{C}}}_{{\rm{chem}}}=-\,\frac{8{{\rm{q}}}^{2}{{\rm{V}}}_{{\rm{film}}}}{{\rm{kT}}}({{\rm{pO}}}_{2}\frac{\partial [{{\rm{V}}}_{{\rm{O}}}^{\mathrm{.}.}]}{\partial {{\rm{pO}}}_{2}}),$$

where q, V_film_, and pO_2_ are the charge of an electron, volume of the film, and partial pressure of oxygen, respectively. In the case of doped CeO_2_ thin films, it represents the formation and annihilation of V_o_, due to the change in oxygen partial pressure. We considered only low pO_2_, because the CeO_2_ films in our case were grown in low pO_2_. For low pO_2_, $[{{\rm{V}}}_{{\rm{o}}}^{\mathrm{}..}]$ could be estimated from the measurement of C_chem_ by utilizing the following equation^44^:

3 $${{\rm{C}}}_{{\rm{chem}}}=\frac{{{\rm{q}}}^{2}{{\rm{V}}}_{{\rm{film}}}}{{\rm{kT}}}({[{{\rm{\Pr }}}_{{\rm{Ce}}}]}_{{\rm{total}}}-2[{{\rm{V}}}_{{\rm{o}}}^{\mathrm{.}.}]),$$

where [Pr_Ce_]_total_ is the total doping concentration of Pr in a CeO_2_ thin film. Equation 3 corresponds to the trivalent dopant in CeO_2_. A similar equation was derived for bivalent dopant at low pO_2_ by replacing the the mass action relation of the trivalent dopant by mass action relation of the bivalent dopant. The mass action or equilibrium equation of Zn-doped CeO_2_ is expressed in Eq. S2 in Supporting information S2. Mass and site conservation reactions are given by^42^

4 $$[{{\rm{Zn}}}_{{\rm{Ce}}}^{^{\prime\prime} }]+[{{\rm{Zn}}}_{{\rm{Ce}}}^{\times }]={[{{\rm{Zn}}}_{{\rm{Ce}}}]}_{{\rm{total}}},$$

where [Zn_Ce_]_total_ is the total doping concentration of Zn in CeO_2_ thin films. For low pO_2_, the electroneutrality and mass balance relation in Equation S4 takes on the following approximation

5 $$[{{\rm{Zn}}}_{{\rm{Ce}}}^{^{\prime\prime} }]=[{{\rm{V}}}_{{\rm{o}}}^{\mathrm{.}.}]-\frac{1}{2}[C{e^{\prime} }_{Ce}]\approx {[{{\rm{Zn}}}_{{\rm{Ce}}}]}_{{\rm{total}}}$$

It is considered that concentration of holes and Ce vacancies are negligibly small and ignored at present situation. Equation 4 can be rewritten as follows by substituting the value of $[{{\rm{Zn}}}_{{\rm{Ce}}}^{^{\prime\prime} }]$ from Eq. 5

6 $$[{{\rm{Zn}}}_{{\rm{Ce}}}^{\times }]\approx {[{{\rm{Zn}}}_{{\rm{Ce}}}]}_{{\rm{total}}}-[{{\rm{V}}}_{{\rm{o}}}^{\mathrm{.}.}]+\,\frac{1}{2}[C{e^{\prime} }_{Ce}]$$

Substituting the values of $[{{\rm{Zn}}}_{{\rm{Ce}}}^{^{\prime\prime} }]$ and $[{{\rm{Zn}}}_{{\rm{Ce}}}^{\times }]$ from Eqs. 5 and 6 in Equation S2 yields

7 $$\frac{{[{{\rm{Zn}}}_{{\rm{Ce}}}]}_{{\rm{total}}}\{{[{{\rm{Zn}}}_{{\rm{Ce}}}]}_{{\rm{total}}}+\,\frac{1}{2}[C{e^{\prime} }_{Ce}]\}{{\rm{pO}}}_{2}^{1/2}}{\{{[{{\rm{Zn}}}_{{\rm{Ce}}}]}_{{\rm{total}}}-[{{\rm{V}}}_{{\rm{o}}}^{\mathrm{.}.}]+\frac{1}{2}[C{e^{\prime} }_{Ce}]\}[{{\rm{O}}}_{{\rm{O}}}^{\times }]}={{\rm{K}}}_{{\rm{Zn}}}$$

Rearranging Eq. 7 yields

8 $${[{{\rm{Zn}}}_{{\rm{Ce}}}]}_{{\rm{total}}}+\frac{1}{2}[C{e^{\prime} }_{Ce}]-[{{\rm{V}}}_{{\rm{o}}}^{\mathrm{.}.}]=\frac{{\{{[{{\rm{Zn}}}_{{\rm{Ce}}}]}_{{\rm{total}}}\}}^{2}+\frac{1}{2}{[{{\rm{Zn}}}_{{\rm{Ce}}}]}_{{\rm{total}}}[C{e^{\prime} }_{Ce}]\}{{\rm{pO}}}_{2}^{1/2}}{{{\rm{K}}}_{{\rm{Zn}}}[{{\rm{O}}}_{{\rm{O}}}^{\times }]}$$

Taking derivative of Eq. 8 w.r.t pO_2_

9 $$\frac{\partial [{{\rm{V}}}_{{\rm{O}}}^{\mathrm{.}.}]}{\partial {{\rm{pO}}}_{2}}=-\frac{1}{2}\frac{{\{{[{{\rm{Zn}}}_{{\rm{Ce}}}]}_{{\rm{total}}}\}}^{2}+\frac{1}{2}{[{{\rm{Zn}}}_{{\rm{Ce}}}]}_{{\rm{total}}}[C{e^{\prime} }_{Ce}]\}{{\rm{pO}}}_{2}^{-1/2}}{{{\rm{K}}}_{{\rm{Zn}}}[{{\rm{O}}}_{{\rm{O}}}^{\times }]}$$

Putting the value of $\frac{\partial [{{\rm{V}}}_{{\rm{O}}}^{\mathrm{.}.}]}{\partial {{\rm{pO}}}_{2}}$ from Eq. 9 in Eq. 3 and rearranging yields

10 $${{\rm{C}}}_{{\rm{chem}}}=\frac{{4{{\rm{q}}}^{2}{{\rm{V}}}_{{\rm{film}}}{[{{\rm{Zn}}}_{{\rm{Ce}}}]}_{{\rm{total}}}\}}^{2}+\frac{1}{2}{[{{\rm{Zn}}}_{{\rm{Ce}}}]}_{{\rm{total}}}[C{e^{\prime} }_{Ce}]\}{{\rm{pO}}}_{2}^{1/2}}{{{\rm{kTK}}}_{{\rm{Zn}}}[{{\rm{O}}}_{{\rm{O}}}^{\times }]}$$

Substituting Eq. 8 in Eq. 10 yields,

11 $${{\rm{C}}}_{{\rm{chem}}}=\frac{4{{\rm{q}}}^{2}{{\rm{V}}}_{{\rm{film}}}}{{\rm{kT}}}({[{{\rm{Zn}}}_{{\rm{Ce}}}]}_{{\rm{total}}}+\frac{1}{2}[C{e^{\prime} }_{Ce}]-[{{\rm{V}}}_{{\rm{o}}}^{\mathrm{.}.}])$$

Equation 11 represents the relationship between the concentration of isolated V_o_ in Zn-doped CeO_2_ films and chemical capacitance extracted by the EIS. If the doping concentration of Zn ([Zn_Ce_]_total_) and the concentration of reduced Ce^3+^ $([C{e^{\prime} }_{Ce}])$ is known in the Zn-doped CeO_2_ thin films, the concentration of V_o_ can be extracted.

[Zn_Ce_]_total_ and $[C{e^{\prime} }_{Ce}]$ can be calculated from XPS data as follows assuming the cross-section of each elemental peak is the same^45^:

13 $${[{{\rm{Zn}}}_{{\rm{Ce}}}]}_{{\rm{total}}}=\frac{{{\rm{A}}}_{{\rm{Zn}}}/{{\rm{S}}}_{{\rm{Zn}}}}{\sum \,{{\rm{A}}}_{{\rm{i}}}/{{\rm{S}}}_{{\rm{i}}}},$$

14 $$[C{e^{\prime} }_{Ce}]=\frac{{A}_{C{e}^{+3}}/{S}_{C{e}^{+3}}}{\sum \,{A}_{i}/{S}_{i}},$$

15 $$\sum {A}_{i}/{S}_{i}=\frac{{A}_{Zn}}{{S}_{Zn}}+\frac{{A}_{C{e}^{+3}}}{{S}_{C{e}^{+3}}}+\frac{{A}_{O2}}{{S}_{O2}},$$

where A_Zn_, ${A}_{C{e}^{+3}}$ and *A*_*O2*_ are the areas of Zn, Ce^3+^ and O_2_ peaks in XPS spectra, respectively, and the *S*_*Zn*_ (31.861), ${S}_{C{e}^{+3}}$ (61.447) and *S*_*O2*_ (2.881) are the atomic sensitivity factors of Zn, Ce^3+^ and O_2_, respectively. The volume of the film was calculated to be 2 cm × 2 cm × 50 nm (length × width × thickness). The calculated values of $[{{\rm{V}}}_{{\rm{o}}}^{\mathrm{}..}]$ for different doping concentrations of Zn is plotted in Fig. 4(c). As can be seen in Fig. 4(c), the concentration of isolated V_o_ increases with the increase in doping concentration which was consistent with the increase in the conductivity of 6ZnCeO_2_ and 14ZnCeO_2._ However, there was a minute increase in the concentration of V_o_ on further increasing the doping concentration from 14% to 24%. As previously explained, in heavily doped samples, the association between V_o_ and dopant becomes strong, and the isolated V_o_ gets trapped in the (*Zn*, *V*_*o*_)^×^ clusters. This phenomenon does not allow V_o_ to increase considerably in heavily doped sample.

**Figure 5:**
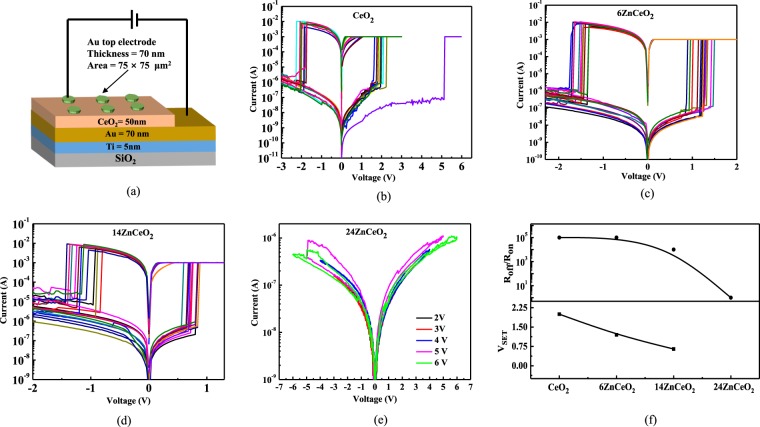
I–V characteristics for (**a**) un-doped CeO2 (**b**) 6ZnCeO_2_ (**c**) 14ZnCeO_2_ (**d**) 24ZnCeO_2_, and (**e**) Plot of variation in R_off_/R_on_ ratio and V_SET_ on increasing the doping concentration.

Figure 5 shows the I–V characteristics for (b) the un-doped CeO_2_ (c) the 6ZnCeO_2_ (d) the 14ZnCeO_2_ (e) the 24ZnCeO_2_, respectively, with (a) the schematic diagram of Au/Zn-doped CeO_2_/Au devices. Figure 5(f) shows the R_off_/R_on_ ratio and V_SET_ on increasing the doping concentration. Both R_off_/R_on_ ratio and V_SET_ decreases on increasing the doping concentration. In order to initiate the resistive switching in undoped CeO_2_, the electrical forming step was applied to the sample. Figure 5(b) presents the electroforming curve and subsequent bipolar resistive switching curves of the CeO_2_ film. The electroforming occurred at 5.2 V. After the electroforming step, the device showed a typical resistive switching behavior with reliable repeatability of the switching cycles.

In the SET process, the device is first driven from the high resistance state (HRS) or the OFF state toward the low resistive state (LRS) or the ON state by applying a positive bias on the top electrode (Au) as shown in Fig. 5(a). The voltage at which the sharp increase in current is observed is termed as V_SET_. When the negative voltage is applied at the top electrode, the process is reversed. This transition of device from LRS to HRS at a particular voltage (V_RESET_), is referred as the RESET process. Without doping in the CeO_2_, the IV curves showed high operating voltage. According to the Raman, XPS, and EIS spectroscopies, which was for the case of the un-doped CeO_2_, the oxygen vacancy level was low. Hence, a large value of voltage was required to induce resistive switching was ascribed to the low level of oxygen vacancy concentration.

**Figure 6:**
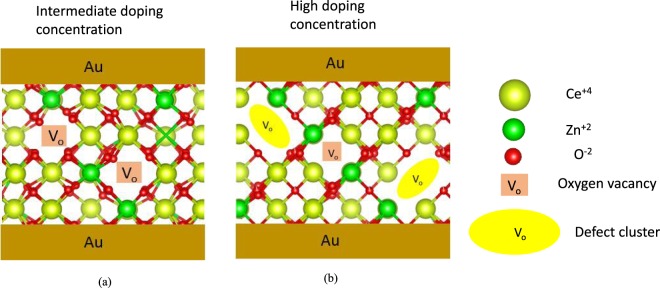
Representation of isolated and cluster defects at various doping levels.

In the doped CeO_2_, the forming step was not necessary since there was already a significant amount of V_o_. Typically, the forming process is introduced to create defects to initiate resistive switching. At low Zn doping concentration, a reduction in V_SET_ was observed as shown in Fig. 5(c), which shows the effect of easy oxygen ionic motion. As a result, lower operating voltage was observed for the 6ZnCeO_2_ device. The on/off ratio up to 10^5^ was maintained for a doping concentration of 6% Zn. The 14% Zn doping concentrations resulted in a more pronounced reduction in operating voltage. However, with this doping range, the on/off ratio was reduced to 10^4^. We interpreted this finding by the increased mobility of the oxygen ions due to increased oxygen vacancies. This result is consistent with the XPS and Raman spectroscopy indicating the increase in Ce^3+^ ions and V_o_ related Raman modes by increasing dopant concentration. By further increasing the doping concentration up to 24%, the resistive switching was diminished. At higher doping concentrations, bulk conductivity was reduced due to decreasing mobility of the isolated V_o_ by the increased number density of the (*Zn*, *V*_*o*_)^×^defect clusters. At low doping concentrations, the isolated V_o_ existed in the CeO_2_ lattice but as the doping concentration increased, the number of isolated V_o_ increased and the association between the defects and V_o_ also occurred and formed neutral or charged clusters. The size and number density of these (*Zn*, *V*_*o*_)^×^ defect clusters increased slightly with the doping concentration. When their number density was small at intermediate doping (Fig. 6(a)), it was energetically more favorable for the oxygen vacancies to rearrange and initiate further reduction in operating voltage at intermediate doping concentrations. At high doping concentrations (Fig. 6(b)), when the size and number density of these clusters increased, these clusters caused hindrance in the mobility of the V_o_. When these V_o_ are trapped by the defect clusters, it makes it difficult for the oxygen ions to hop over the vacancies. Hence, the mobility of the oxygen ions will be reduced. Figure 5(f) shows the results for the V_SET_ and the R_off_/R_on_ ratio for the different Zn concentrations. A maximum in the V_SET_ was observed for the device without doping. The V_SET_ was minimum at an intermediate Zn doping concentration. At high Zn doping concentrations, resistive switching was diminished. Similarly, a decrease in the R_off_/R_on_ ratio was observed for the intermediate Zn doping concentrations. Comparing these results to the ionic conductivity, we demonstrated that there is a connection between the ionic conductivity of the oxide and the switching characteristics such as V_SET_ and R_off_/R_on_ in resistive switching devices. This reduction in the R_off_/R_on_ ratio and the V_SET_ was ascribed to the increase in ionic conductivity by increasing the doping concentration from 6% to 14%. The association of ionic conductivity of the Zn-doped CeO_2_ with different Zn concentrations is explained in relation to the impedance spectroscopy analysis. The device to device variation of undoped CeO2, 6ZnCeO2, and 14ZnCeO2 is given in Figure S5. The statistical data indicate that there is no significant variation in the V_SET_.

**Figure 7:**
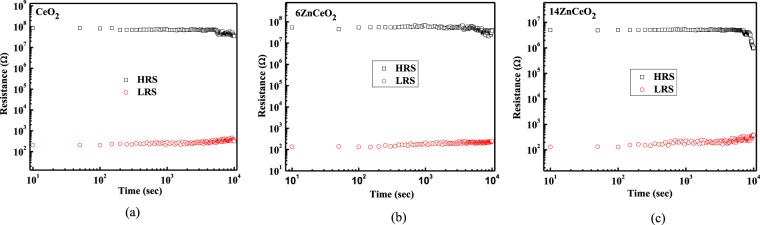
Retention data of (**a**) un-doped CeO_2_ (**b**) 6ZnCeO_2_, and (**c**) 14ZnCeO_2_ devices in the LRS (hollow circles) and HRS (hollow squares) at room temperature.

The retention measurement results of the un-doped CeO_2_, 6ZnCeO_2_, and 14ZnCeO_2_ devices at room temperature by applying reading bias of + 0.2 V are shown in Fig. 7(a–c). The R_off_/R_on_ ratio was maintained at 10^5^ with no significant degradation in resistance after 10^4^ sec in the un-doped CeO_2_, and 6ZnCeO_2_. However, the on/off ratio was reduced to 10^4^ in the 14ZnCeO_2_ device. This decrease in the R_off_/R_on_ ratio was associated with the increase in ionic conductivity of oxygen ions by increasing the doping concentration from a 6% to a 14% doping concentration as illustrated in Fig. 5(f), which caused low R_off_/R_on_ ratio. Zn-doped CeO_2_ devices with the intermediate doping level showed a great potential for nonvolatile memory applications with the low V_SET_/V_RESET_, high R_off_/R_on_ ratio, and good retention characteristics.

The endurance data for un-doped CeO_2_, 6ZnCeO_2_, and 14ZnCeO_2_ are given in Figure S7. The R_off_/R_on_ ratio of CeO_2_ and 6ZnCeO_2_ devices was maintained at 10^5^ without any significant degradation up to 250 cycles. Although the on/off ratio of 14ZnCeO_2_ device was reduced to 10^4^, no degradation of R_off_/R_on_ ratio was observed.

## Conclusions

Zn-doped CeO_2_ active layer is utilized for resistive switching. Raman spectroscopy is employed to study the structural modification introduced by the dopant in the host lattice of CeO_2_. An increase in FWHM of the characteristics peak of CeO_2_ (460 cm^−1^) and the enhancement of the defect related peak (560 cm^−1^) confirms the uniform doping of Zn in CeO_2_ and the existence of (*Zn*, *V*_*o*_)^×^ defect clusters in Zn-doped CeO_2_. Increased doping of Zn in CeO_2_ leads to the formation of more oxygen vacancies in Zn doped CeO_2_. Increase in oxygen vacancies with an increasing doping concentration results in reduction of operating voltage in 6ZnCeO_2_ and 14ZnCeO_2_ devices as compared to a CeO_2_ device. A further increase in the doping concentration leads to the diminishing of resistive switching behavior in a 24CZnO device. This behavior is explained by the increased number density of (*Zn*, *V*_*o*_)^×^ defect clusters which decrease the mobility of V_o_ in the highly doped CeO_2_.

## Supplementary information

Supplementary Information
