# Supplementary material for: Tuning of ionic mobility to improve the resistive switching behavior of Zn-doped CeO2
Source: Sci Rep. 2019 Dec 18;9:19387. doi: 10.1038/s41598-019-55716-4 (PMC6920484; doi:10.1038/s41598-019-55716-4)
Supplement: Supplementary file 1 — Supplementary Information [file 41598_2019_55716_MOESM1_ESM.docx]

**Supporting information**

**Tuning of ionic mobility to improve the resistive switching behavior of Zn-doped CeO_2_**

Shania Rehman^1^, Honggyun Kim^1^, Muhammad Farooq Khan^1^, Ji-Hyun Hur^1^_,_ Anthony D. Lee^2^ and Deok-kee Kim^1*^

^1^Department of Electrical Engineering, Sejong University, Seoul 05006, Republic of Korea

^2^Department of Mechanical Engineering Technology, Farmingdale State College, Farmingdale, New York 11735, USA

* deokkeekim@sejong.ac.kr

**Supporting information S1: Defect relations for doping in CeO_2_**

The doping effect of trivalent or bivalent dopant become clear by considering the following defect reactions, using $\mathrm{kro}\ddot{g}\mathrm{er}$-vink notation [^s1^](#_ENREF_1).

$$M_{2}O_{3}\underset{\to}{{CeO}_{2}}{2M}_{Ce}^{'}+{3O}_{o}^{x}+V_{o}^{\cdot\cdot}$$

$MO\underset{\to}{{CeO}_{2}}M_{Ce}^{''}+O_{o}^{x}+V_{o}^{\cdot\cdot}$,

where ${2M}_{Ce}^{'}$, $M_{Ce}^{''}$, $O_{o}^{\times}$, and $V_{o}^{..}$ are trivalent and bivalent dopants on cerium sites, oxygen on oxygen site, and oxygen vacancies with +2 charge, respectively. In superscripts, ×, prime and dot represent neutral, -1 charge and +1 charge, respectively. As it can be seen for the case of trivalent dopant, for every two dopant atoms, one oxygen vacancy is created. For bivalent doping, one oxygen vacancy is created for every single dopant.

**Supporting information S2: Defect relations of Zn-doped CeO_2_**

In $\mathrm{kro}\ddot{g}\mathrm{er}$-vink notation, the defect reaction for the creation of V_o_ by ZnO doping into CeO_2_ can be written as:

$\mathrm{Zn}_{\mathrm{Ce}}^{\times}+ O_{o}^{\times}\to\mathrm{Zn}_{\mathrm{Ce}}^{''}+ V_{o}^{..}+\frac{1}{2}O_{2}$, (S1)

where $\mathrm{Zn}_{\mathrm{Ce}}^{\times}$, $\mathrm{Zn}_{\mathrm{Ce}}^{''}$, $O_{o}^{\times}$, and $V_{o}^{..}$ are Zn^+4^ and Zn^+2^ on cerium sites, oxygen on oxygen site, and oxygen vacancies with +2 charge, respectively. In superscripts, ×, prime and dot represent neutral, -1 charge and +1 charge, respectively. The concentration of V_o_ and dopant are related by the equilibrium constant K_Zn_ of mass action or equilibrium equation corresponding to defect reaction in Eq. S1.

$\frac{\left[ \mathrm{Zn}_{\mathrm{Ce}}^{''} \right]\left[ V_{o}^{..} \right]\mathrm{pO}_{2}^{\frac{1}{2}}}{\left[ \mathrm{Zn}_{\mathrm{Ce}}^{\times} \right]\left[ O_{O}^{\times} \right]}=K_{Zn,}$ (S2)

where $\mathrm{pO}_{2}^{\frac{1}{2}}$ is the partial pressure of oxygen and the equilibrium constant K_Zn_ of mass action or equilibrium equation is expressed as ^S2^

$K_{\mathrm{Zn}}= k_{\mathrm{Zn}}^{o}\exp\left( \frac{{-H}_{\mathrm{Zn}}}{\mathrm{kT}} \right)$, (S3)

where $k_{\mathrm{Zn}}^{o}$, $H_{\mathrm{Zn}}$, k, and T are pre-exponential term, enthalpy of the reaction, Boltzmann constant, and temperature, respectively.

For determining the concentration of oxygen vacancies, $\mathrm{Zn}_{\mathrm{Ce}}^{''}$ and $\mathrm{pO}_{2}^{\frac{1}{2}}$ are two most decisive parameters in Eq. S2. As more Ce^+4^ atoms of host lattice are replaced with Zn^+2^ cations, more O vacancy will be formed. The $\mathrm{pO}_{2}^{\frac{1}{2}}$ is intentionally kept low with Ar:O_2_ ratio being 87.5:12.5 to intrinsically introduce some oxygen vacancies in CeO_2_. However, the role of $\mathrm{pO}_{2}^{\frac{1}{2}}$ is eliminated from Eq. 11 in manuscript for calculating the V_o_ concentration because, in the present study, we do not consider the effect of intrinsic V_o,_ but we are more interested in the modulation of extrinsic V_o_ created by Zn doping.

The electroneutrality equation for Zn doped CeO2 is expressed as ^S3^

$2\left[ V_{o}^{..} \right]=2\left[ {Zn}_{Ce}^{''} \right]+\left[ {Ce}_{Ce}^{'} \right]+4\left[ V_{Ce}^{'''} \right]+3\left[ \left( Zn, {Ce}_{Ce} \right)^{'''} \right],$ (S4)

where ${[Ce}_{Ce}^{'}],$ $[V_{Ce}^{'''}]$ and $[\left( Zn, {Ce}_{Ce} \right)^{'''}]$ are the concentration of reduced Ce^+3^ ions, Ce vacancies and defect associates of dopant and cerium ions in Zn-doped CeO_2_ thin films. ${-H}_{\mathrm{Zn}}$

The formation reaction for these associated defects ${(Zn, V_{o})}^{\times}$ between bivalent dopant (Zn^+2^ in the present case) and V_o_ is written as ^S3^

${Zn}_{Ce}^{''}+ V_{o}^{..}={(Zn, V_{o})}^{\times}$ (S5)

The concentration of these associated defects can be obtained as a function of equilibrium constant K_AV_ [^3^](#_ENREF_3), by using equilibrium equation in Eq. S5.

$K_{\mathrm{Av}}= \frac{\left[ \left( Zn, V_{o} \right)^{\times} \right]}{\left[ \mathrm{Zn}_{\mathrm{Ce}}^{''} \right]\left[ V_{o}^{..} \right]}$ (S6)

**Supporting information S3: X-ray photoelectron spectroscopy**

**Figure S3(a):**

Figure S3(a): XPS Survey spectra for undoped and Zn-doped CeO_2_

**Figure S3(b):**

Figure S3(b): High resolution XPS spectra of O1s in undoped and Zn-doped CeO_2_

**Figure S4**

Figure S4: Plot of parameters extracted from data fitting of experimentally obtained EIS spectra with equivalent circuit, for undoped and doped CeO_2_ with different doping levels.

**Figure S5**

Figure S5: Device to device variability of undoped and Zn doped CeO2 thin films

**Figure S6**

(c)

(b)

(a)

Figure S6: Endurance data of (a) un-doped CeO_2_ (b) 6ZnCeO_2_, and (c) 14ZnCeO_2_ devices in the LRS (hollow squares) and HRS (hollow circles) at room temperature.

**References:**

S1 Li, Z.-P., Mori, T., Zou, J. & Drennan, J. Defects clustering and ordering in di-and trivalently doped ceria. *Materials Research Bulletin* **48**, 807-812 (2013).

S2 Yang, N. *et al.* Role of associated defects in oxygen ion conduction and surface exchange reaction for epitaxial samaria-doped ceria thin films as catalytic coatings. *ACS applied materials & interfaces* **8**, 14613-14621 (2016).

S3 Pijolat, M., Prin, M., Soustelle, M., Touret, O. & Nortier, P. Thermal stability of doped ceria: experiment and modelling. *Journal of the Chemical Society, Faraday Transactions* **91**, 3941-3948 (1995).
